# Associations of Coffee and Tea Consumption on Neural Network Connectivity: Unveiling the Role of Genetic Factors in Alzheimer’s Disease Risk

**DOI:** 10.3390/nu16244303

**Published:** 2024-12-13

**Authors:** Tianqi Li, Mohammad Fili, Parvin Mohammadiarvejeh, Alice Dawson, Guiping Hu, Auriel A. Willette

**Affiliations:** 1 Genetics and Genomics Program, Iowa State University, Ames, IA 50011, USA; tianqili@iastate.edu; 2 School of Industrial Engineering and Management, Oklahoma State University, Stillwater, OK 74078, USA; mohammad.fili@okstate.edu (M.F.); pmohamm@iastate.edu (P.M.); guiping.hu@okstate.edu (G.H.); 3 Department of Industrial and Manufacturing Systems Engineering, Iowa State University, Ames, IA 50011, USA; 4 Chestnut Health Systems, Lighthouse Institute, Chicago, IL 60610, USA; a.l.dawson.sciences@gmail.com; 5 Department of Neurology, Rutgers University, New Brunswick, NJ 08854, USA

**Keywords:** aging, functional connectivity, diet, cognitive impairment

## Abstract

Background: Coffee and tea are widely consumed beverages, but their long-term effects on cognitive function and aging remain largely unexplored. Lifestyle interventions, particularly dietary habits, offer promising strategies for enhancing cognitive performance and preventing cognitive decline. Methods: This study utilized data from the UK Biobank cohort (*n* = 12,025) to examine the associations between filtered coffee, green tea, and standard tea consumption and neural network functional connectivity across seven resting-state networks. We focused on networks spanning prefrontal and occipital areas that are linked to complex cognitive and behavioral functions. Linear mixed models were used to assess the main effects of coffee and tea consumption, as well as their interactions with Apolipoprotein E (APOE) genetic risk—the strongest genetic risk factor for Alzheimer’s disease (AD). Results: Higher filtered coffee consumption was associated with increased functional connectivity in several networks, including Motor Execution, Sensorimotor, Fronto-Cingular, and a Prefrontal + ‘What’ Pathway Network. Similarly, greater green tea intake was associated with enhanced connectivity in the Extrastriate Visual and Primary Visual Networks. In contrast, higher standard tea consumption was linked to reduced connectivity in networks such as Memory Consolidation, Motor Execution, Fronto-Cingular, and the “What” Pathway + Prefrontal Network. The APOE4 genotype and family history of AD influenced the relationship between coffee intake and connectivity in the Memory Consolidation Network. Additionally, the APOE4 genotype modified the association between standard tea consumption and connectivity in the Sensorimotor Network. Conclusions: The distinct patterns of association between coffee, green tea, and standard tea consumption and resting-state brain activity may provide insights into AD-related brain changes. The APOE4 genotype, in particular, appears to play a significant role in modulating these relationships. These findings enhance our knowledge of how commonly consumed beverages may influence cognitive function and potentially AD risk among older adults.

## 1. Introduction

Alzheimer’s disease (AD) is by far the most common dementia diagnosis, accounting for roughly 80% of cases, and is becoming one of the most financially burdensome and, thereby, one of the most prioritized health issues of study in the 21st century [1]. As the population ages in the US, the prevalence of Alzheimer’s disease is expected to increase dramatically. In 2022, an estimated 6.5 million older Americans were living with Alzheimer’s disease, with nearly three-quarters of these individuals being over 75 years old [2]. This number is projected to reach 13.8 million by 2060, leading to substantial strain on the economy [2,3]. Due to this impending health and economic crisis, it is crucial that the scientific community develop a thorough understanding of what factors influence AD risk, in particular regarding lifestyle and dietary choices [4].

Diet has emerged as a crucial risk factor for all-cause dementia, particularly AD [5,6]. In our earlier work, we found that the intake of specific nutrients by cognitively unimpaired adults—including B vitamins and certain foods like red wine, milk, and cheese—correlated with alterations in resting-state functional connectivity that are sensitive to AD [7,8]. Notably, these connectivity fluctuations are influenced by familial AD history and Apolipoprotein E ε4 (APOE4) genotypes, which are the strongest genetic risk factors for AD [9]. Building upon these findings, there is a need to explore other categories of nutritional compounds as potential biomarkers for tracking resting-state functional connectivity patterns, like functional magnetic resonance imaging (fMRI) [10,11,12]. Studies employing Independent Component Analysis (ICA) have uncovered unique connectivity patterns in individuals diagnosed with Mild Cognitive Impairment (MCI) or AD [13]. This approach may provide valuable insights into the complex interplay between diet, genetics, and AD risk.

Globally, a significant portion of the population consumes coffee and tea on a regular basis, making them among the most popular beverages worldwide [14,15]. Besides caffeine, these drinks contain numerous bioactive substances, specifically phenolic compounds, which have garnered attention for their potential health effects. Furthermore, some bioactive components in tea and coffee exhibit antioxidant, anti-inflammatory, and antimicrobial properties, suggesting the potential to mitigate various non-communicable chronic conditions [15,16,17].

However, the way in which the regular consumption of coffee and tea impacts human health remains a subject of debate. Observational studies exploring the relationship between the consumption of these beverages and dementia risk have yielded conflicting results [18,19]. Some studies suggest that increased intake of coffee and tea (separately or combined) might be associated with reduced dementia risk [20,21]. Conversely, other studies have not indicated a significant association between coffee and cognitive decline or dementia progression [22,23]. Similar inconsistencies have been observed in studies of tea consumption [24,25].

Thus, we explored the associations of tea and coffee consumption on cognition and long-term risk for AD, given their widespread popularity and potential neurological relationships. To this end, we examined data from UK Biobank participants. Our investigation centered on analyzing the association between green tea, standard black tea, and filtered coffee intake and the extent of neural network functional connectivity across several key networks implicated in sensory, motor, and cognitive processing. Specifically, we focused on the following networks: the Primary Visual Network and Extrastriate Visual Network, the Memory Consolidation Network, the Motor Execution Network and Sensorimotor Network, the Fronto-Cingular Network, and the Prefrontal plus ‘What’ Pathway Network. These networks play pivotal roles in maintaining cognitive function and are highly relevant to AD [26,27].

The Memory Consolidation Network, for example, is critical for the retrieval and storage of long-term memories [26], a function that is frequently impaired in early AD. Similarly, the Fronto-Cingular Network, involving the anterior cingulate cortex, is associated with cognitive control and emotional regulation [27], which are disrupted in individuals with AD. The Primary Visual and Extrastriate Visual Networks, located in the occipital lobe [28], are essential for visual processing and integration, and their dysfunction may contribute to the visuospatial impairments observed in AD. Furthermore, networks such as the Motor Execution and Sensorimotor Networks are integral to motor planning and body awareness [29], while the Prefrontal plus ‘What’ Pathway Network supports decision-making and object recognition [30]. These neural systems collectively provide a comprehensive framework for understanding how coffee and tea consumption may influence brain connectivity and cognitive resilience. This analysis was further stratified by genetic AD risk factors, such as APOE4 status and the family history of AD, as these genetic predispositions are strongly associated with altered connectivity patterns in these networks [31,32]. Such stratification allowed us to examine potential interactions between dietary habits and genetic risk factors, contributing to a more nuanced understanding of AD-related brain changes.

## 2. Materials and Methods

### 2.1. Cohort and Participants

The UK Biobank, a large-scale prospective study, encompasses nearly 500,000 participants. These individuals, aged between 40 and 70 at the time of enrollment, were recruited from 22 assessment centers across the United Kingdom [33]. In this study, we examined a subset of 12,025 participants who met the criteria. Specifically, we included only those participants without diagnosis following conditions (with ICD-10 code), central nervous system disorders (G00–G99), cerebrovascular ailments (I60–I69), and mental or behavioral issues (F00–F99) [7]. This cohort provided comprehensive data, including genetic information, resting-state functional magnetic resonance imaging (rsfMRI) scans, self-reported tea and coffee consumption patterns, and demographic details.

### 2.2. Resting-State fMRI

Imaging data were conducted at three sites (Reading, Newcastle, and Manchester) using Siemens Skyra 3T scanners equipped with 32-channel RF receiver head coils (Siemens Medical Solutions, Erlangen, Germany) [34]. MRI data collection began in 2014, with ongoing longitudinal assessments [35]. Participants were instructed to maintain open eyes, focus on a fixation cross, and avoid specific thoughts during the 6 min and 10 s scans, which captured 490 images. More specific details of these methodologies can be found in our previous study [36]. Preprocessing and quality control procedures are detailed in the UK Biobank white papers (https://biobank.ctsu.ox.ac.uk/crystal/crystal/docs/brain_mri.pdf (4 December 2024)). Twenty-one non-noise Independent Components (ICs) were identified to represent resting-state neural networks [37]. These ICs can be visualized online using Papaya viewer: https://www.fmrib.ox.ac.uk/ukbiobank/group_means/rfMRI_ICA_d25_good_nodes.html (7 November 2024).

To identify intrinsic functional connectivity (iFC) networks, we applied group-level Independent Component Analysis (ICA) using the Group ICA Toolbox [38,39]. This method decomposes resting-state fMRI data into spatially Independent Components (ICs), which correspond to functional networks [40]. The process began with two stages of data reduction: first, principal components (PCs) were generated for each subject and session, retaining 99% of the variance. Then, these PCs were further reduced to ICs, capturing 88.66% of the total variance [39]. The Infomax ICA algorithm was employed to extract ICs, and the decomposition was repeated three times with the ICASSO algorithm to improve reliability through clustering [39,41]. Individual ICs for each participant were reconstructed using the GICA3 algorithm, preserving subject-specific variability [37]. To identify relevant networks, the ICs were compared to spatial templates and ranked based on their correlation, with expert review (AAW) confirming alignment with known functional networks. Finally, spatial maps were converted to Z-scores, and mean activation levels were calculated for each participant, with motion parameters included as nuisance regressors in the subsequent analyses. An author (AAW) reviewed the activation maps and identified the neural networks, as our previous research details [36].

To reduce Type I errors, our current study focused on seven networks associated with sensory integration, cognitive processing, and Motor Execution functions (refer to Supplemental Table S1). Briefly, these seven networks were the Extrastriate Visual Network (IC 4); Memory Consolidation Network (IC 7); Primary Visual Network (IC 18); Motor Execution Network (IC 11); Sensorimotor Network (IC 12); Fronto-Cingular Network (IC 14); and the Prefrontal + ‘What’ Pathway Network (IC 21).

These networks were chosen based on their established roles in maintaining functional independence and their relevance to AD. The Memory Consolidation Network is essential for encoding, retrieving, and storing long-term memories, which are processes that are typically impaired in the early stages of AD. The Extrastriate Visual Network and Primary Visual Network play critical roles in visual processing and integration, which are necessary for visuospatial abilities often compromised in AD. The Motor Execution Network and Sensorimotor Network support motor planning, coordination, and body awareness, all of which are vital for physical independence and quality of life. The Fronto-Cingular Network is associated with cognitive control, emotional regulation, and error monitoring, which are increasingly disrupted as AD progresses. Finally, the Prefrontal + ‘What’ Pathway Network facilitates decision-making and object recognition, which are key cognitive functions that decline with disease progression.

By focusing on these seven networks, our study specifically targeted neural systems that are crucial to sensory, motor, and cognitive functions, enabling us to investigate potential pathways through which dietary factors, such as tea and coffee consumption, might influence AD risk. This targeted approach also minimized the likelihood of false positives and ensured that the analysis remained aligned with the study’s primary objective of understanding the intrinsic functional connectivity patterns most relevant to AD-related changes.

### 2.3. Genetic Factors—APOE and AD Family History

Genetic analysis was conducted using either the UK BiLEVE Axiom or the UK Biobank Axiom array [42]. APOE haplotype isoforms (ε2, ε3, and ε4) were determined using SNPs rs429358 and rs7412. Participants were classified as ε4 carriers if they possessed at least one ε4 allele (ε2/ε4, ε3/ε4, or ε4/ε4), while those without any ε4 alleles (ε2/ε2, ε2/ε3, or ε3/ε3) were designated as non-carriers. To ascertain Alzheimer’s disease (AD) through family history [7,8,36], we relied on self-reported data collected via a touchscreen questionnaire. Participants responded to the query, “Has/did your father/mother ever suffer from:”, followed by a list of chronic conditions, including “Alzheimer’s disease/dementia.” This information was used to classify participants based on the presence or absence of AD in their family history [7].

### 2.4. Covariates

Covariates included baseline age (in years), biological sex (male or female), alcohol consumption status (never, previous, or current drinker), smoking habits (never, former, or current smoker) [43], body mass index (BMI), and the Townsend deprivation index for socio-economic stratification. The Townsend index, a measure of relative social deprivation, was computed prior to each participant’s enrollment in the UK Biobank. This index, derived from national census data on output areas, assigns scores to participants based on their residential postcode, which acts as a proxy for socio-economic status [44].

### 2.5. Tea and Coffee Consumption

Dietary data, including beverage consumption, were collected using an interactive touchscreen questionnaire. This assessment employed a 24 h dietary recall method, a widely recognized approach in nutritional epidemiology for the effective capture of detailed dietary information [45]. Participants were asked to report their consumption of various food and drink items from the previous day.

Specifically, coffee intake (UKB Data-field ID: 100270) was assessed with the question: “How many cups/mugs of filter/americano/cafetiere coffee did you drink yesterday?” Tea consumption (UKB Data-field ID: 100400 and ID: 100420) was evaluated using a similar approach, with participants responding to the question: “How many cups/mugs of standard tea (e.g., Tetley, PG Tips, Assam, Darjeeling)/green tea did you drink yesterday?” Broadly, this would be different types of black tea in the UK.

These questions were designed to capture typical consumption patterns of the most commonly consumed caffeinated beverages in the United Kingdom. By specifying “standard tea” and providing examples, the questionnaire aimed to standardize responses and reduce potential misclassification. Similarly, the coffee question focused on specific preparation methods (filter, americano, cafetiere) to enhance the precision of the data collected. The full questionnaire, which includes a comprehensive range of all dietary items assessed, is available for review at https://biobank.ctsu.ox.ac.uk/crystal/crystal/docs/DietWebQ.pdf (accessed on 11 August 2024).

### 2.6. Statistical Analyses

Data preparation and statistical analyses were performed using R (version 4.4.1) (RStudio 2024.09.1+394, Posit Software, Boston, MA, USA). We used linear mixed models (LMMs) to examine the relationships between dietary predictors (coffee, herbal tea, standard tea consumption) and each resting-state Independent Component (IC), with each IC representing a specific neural network. The outcome of interest was a Z-score value for the intrinsic functional connectivity of the network relative to other adults. These Z-scores were derived from Independent Component Analysis (ICA), as detailed earlier in the methodology.

The fixed effects in our models included coffee, standard tea, or herbal tea consumption as continuous predictors. Covariates included age, sex, body mass index (BMI), smoking status, alcohol consumption, and socioeconomic status (e.g., Townsend deprivation index). These covariates were chosen based on the existing literature that highlights their potential influence on brain connectivity and cognitive functions. Interaction terms were incorporated to explore whether genetic and familial factors, such as the family history of Alzheimer’s disease (binary: Yes/No) and APOE4 status (binary: positive/negative) both moderated the relationship between dietary predictors and connectivity. Random Effects of the participants’ examination centers were included to account for between-subject variability.

The analysis proceeded in two stages. First, we examined the main effects of coffee, standard tea, or herbal tea consumption on each neural network’s Z-score while adjusting for all covariates. Second, interaction models were constructed to test whether the APOE4 genotype or family history of Alzheimer’s disease influenced these relationships. For instance, models assessed how APOE4 status moderated the effect of coffee consumption on network connectivity or whether the family history of AD interacted with tea consumption to influence connectivity patterns. These models allowed us to capture potential individual differences in the dietary effects on neural connectivity.

All statistical analyses were conducted in R using the lme4 and lmerTest packages for mixed-effects modeling. Interaction effects and visualizations were generated using the ggplot2 packages. Detailed R code, including steps for data preprocessing and model specification, are provided as a supplementary document.

In our analysis, we used an Alpha value of 0.05 (i.e., *p* < 0.05) for the main effects. However, a more lenient Alpha value of 0.10 was used for interaction analyses due to lower statistical power. This approach aligns with recommendations for balancing Type I and Type II error rates in complex models with large data sets [46,47]. To mitigate the risk of type I errors due to multiple comparisons, we initially conducted omnibus multivariate analyses of covariance (MANCOVA) [48]. This preliminary step indicates if an overall effect is significant; for those effects found to be significant, we conducted follow-up comparisons to see exactly which tests drove this overall effect. Given this omnibus testing, the actual versus predicted Alpha remains roughly at 0.05 (*p* < 0.05), which maintains the robustness and reliability of the analytic strategy.

## 3. Results

### 3.1. Demographics and Data Summaries

The sample (*n* = 12,025) included data from predominantly women (52.54%, or n_women_ = 6318) with a mean age of 55.07 y. The full demographic profile and initial characteristics of the study participants are presented in Table 1. A comprehensive list and detailed descriptions of the seven neural networks associated with cognitive and emotional processes, excluding noise-derived components, can be found in Supplementary Table S1.

### 3.2. Main Effects

**Figure 1:**
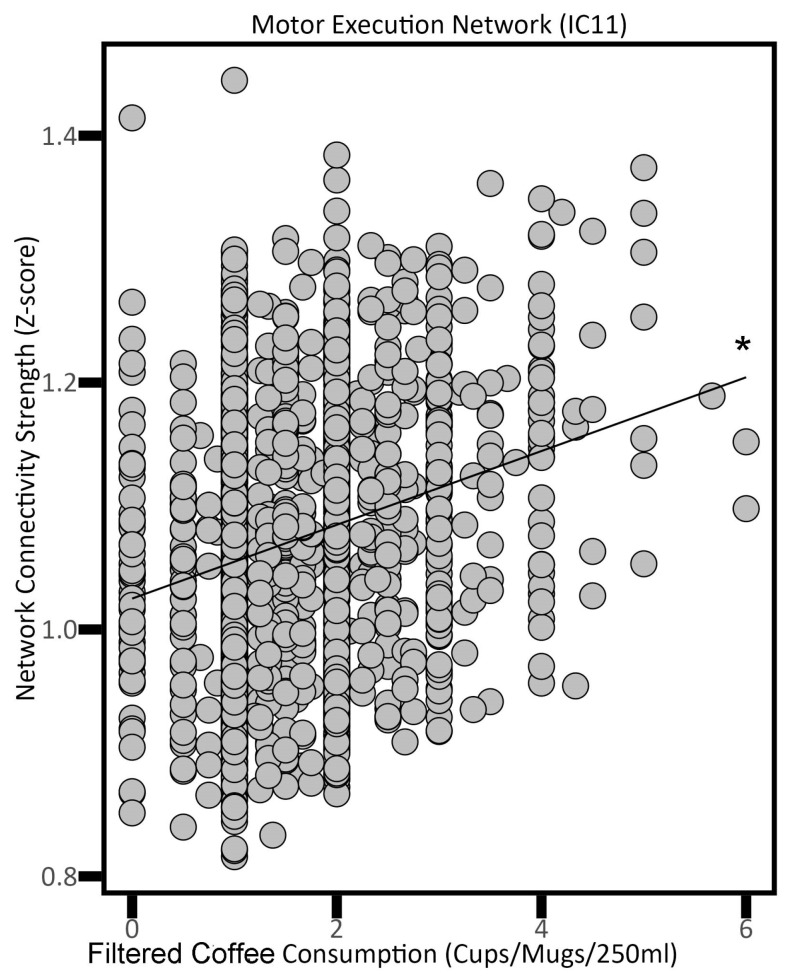
The association between filtered coffee consumption and the Motor Execution Network (i.e., neural network activity) in adults. * *p* < 0.05.

The omnibus test for the main effect of coffee consumption across all networks was non-significant. Increased coffee intake showed positive correlations with functional connectivity across several neural networks. These networks included the Motor Execution Network (IC 11; *p* = 0.0471), the Sensorimotor Network (IC 12; *p* = 0.0011), the Fronto-Cingular Network (IC 14; *p* = 0.0037), and the Prefrontal + ‘What’ Pathway Network (IC 21; *p* = 0.0340) (Table 2). Figure 1 represents an example of this relationship between coffee consumption and functional connectivity in the Motor Execution Network (i.e., IC 11).

**Figure 2:**
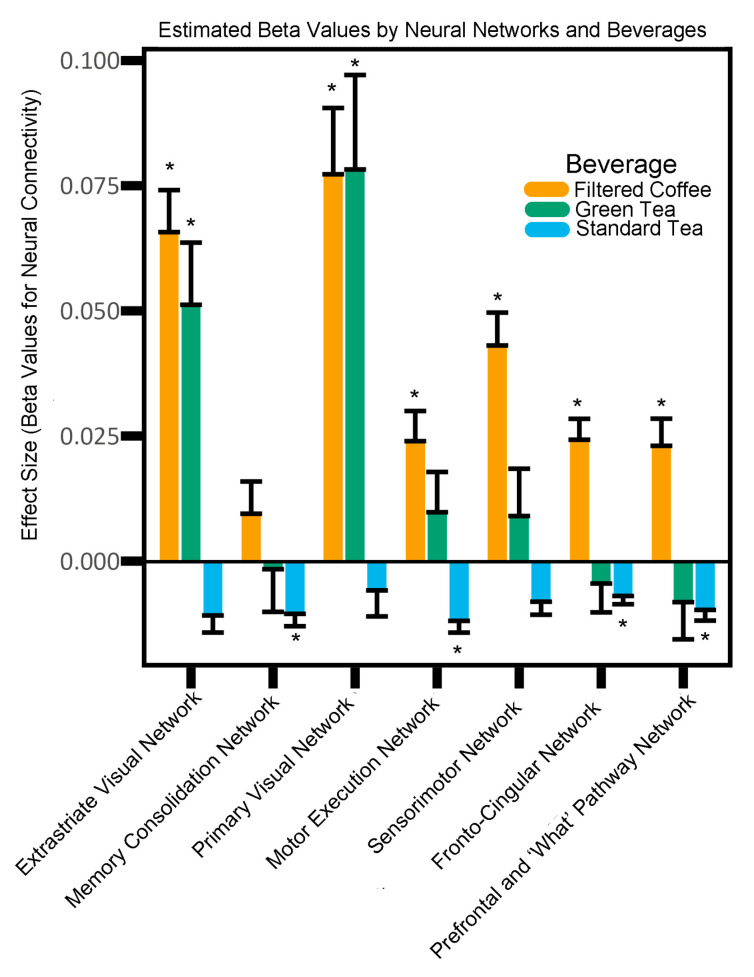
The effect size (i.e., estimated Beta values) for neural network intrinsic functional connectivity in a given network. * *p* < 0.05.

More green tea consumption is associated with a greater Extrastriate Visual Network (IC 4; *p* = 0.0396) and Primary Visual Network (IC 8; *p* = 0.0385) connectivity (Table 2, Figure 2).

**Figure 3:**
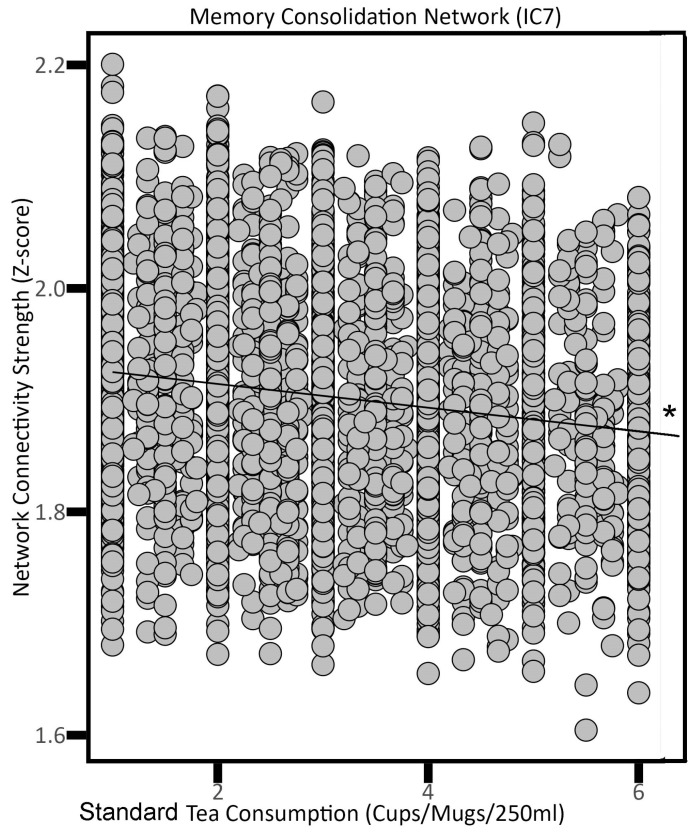
The association between standard tea consumption and the Memory Consolidation Network (i.e., neural network activity) in adults. * *p* < 0.05.

Conversely, higher standard tea consumption was related to decreased connectivity in the Memory Consolidation Network (IC 7; *p* = 0.0329), Motor Execution Network (IC 11; β = −0.0119, *p* = 0.0111), Fronto-Cingular Network (IC 14; *p* = 0.0343), and the Prefrontal + ‘What’ Pathway Network (IC 21; *p* = 0.0227) (Table 2). Figure 3 illustrates an example between standard tea consumption and network strength in the Memory Consolidation Network (i.e., IC 7).

### 3.3. Coffee Consumption: Interactions with APOE4 Status and Family History

The association between coffee intake and neural connectivity varies based on genetic and familial risk factors. For APOE4 status and the Memory Consolidation Network (i.e., IC 7), increased coffee consumption was associated with more functional connectivity in non-APOE4 carriers (β = 0.0302, SE = 0.0026, *p* < 0.001) in contrast to less functional connectivity in APOE4 carriers (β = −0.0313, SE = 0.0043, *p* < 0.001).

**Figure 4:**
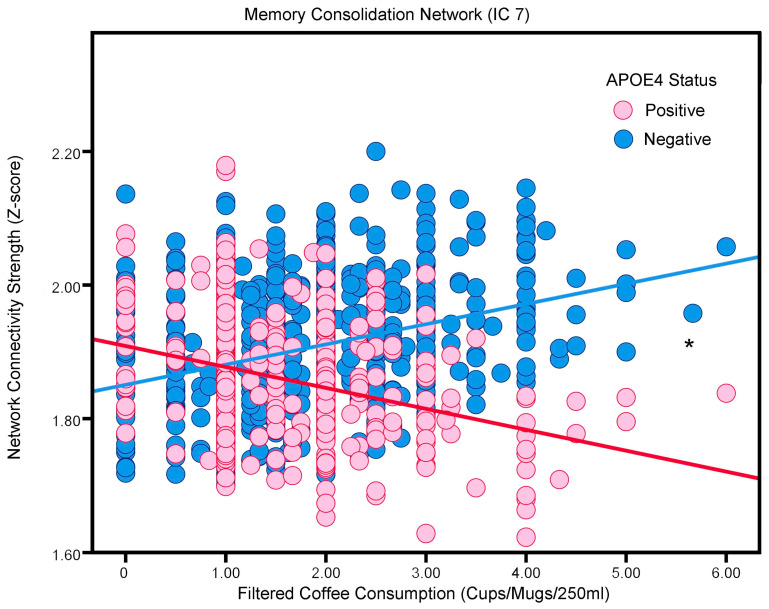
The association between filtered coffee and the Memory Consolidation Network (i.e., neural network activity) in adults without or with the APOE4 allele (“positive”; “negative”). Blue and red, respectively, represent APOE4-negative and APOE4-positive participants. * *p* < 0.05.

A similar pattern emerged when considering the family history of AD. Participants without familial AD history demonstrated more Memory Consolidation Network connectivity with greater coffee consumption (IC 7; β = 0.0351, SE = 0.0029, *p* < 0.001). Conversely, those with a family history of AD exhibited an inverse relationship between functional network connectivity; as coffee intake increased, the functional connectivity of this network decreased (IC 7; β = −0.0330, SE = 0.0037, *p* < 0.001). These findings are visually represented in Figure 4 and Table 3.

### 3.4. Standard Tea Consumption: Interactions with APOE4 Status

**Figure 5:**
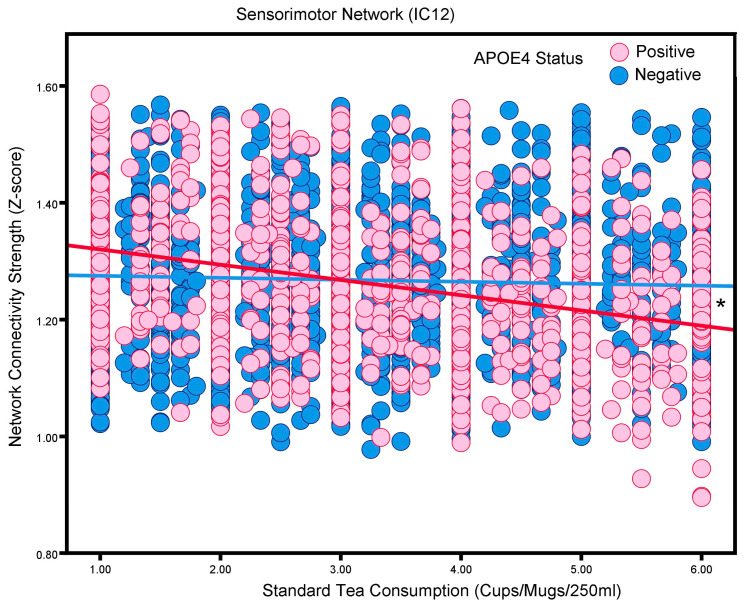
The association between standard tea consumption and the Sensorimotor Network (i.e., neural network activity) in adults without or with the APOE4 allele (“positive”; “negative”). Blue and red, respectively, represent APOE4-negative and APOE4-positive participants. * *p* < 0.05.

APOE4 status also modulated the relationship between standard tea consumption and neural network connectivity. APOE4 carriers showed a negative association between standard tea intake and connectivity in the Memory Consolidation Network (IC 7; β = −0.0261, SE = 0.0054, *p* < 0.001). However, interestingly, no significant correlation was observed between standard tea consumption and network connectivity in APOE4 non-carriers. This result is illustrated in Figure 5 and Table 3.

## 4. Discussion

Coffee and tea, as widely consumed beverages worldwide, continue to intrigue researchers due to their bioactive compounds [14,15]. Besides caffeine, these beverages contain various other bioactive substances, particularly phenolic compounds, which have garnered interest from the scientific community for their effects on oxidative stress and their anti-inflammatory effects [49,50]. This study explored the relationships between coffee and tea consumption and functional connectivity within neural networks, particularly those involving sensory integration, cognitive processing, and Motor Execution. Our analysis considered the potential influences of APOE4 genotypes and familial AD history. The results reveal distinct patterns of neural connectivity associated with coffee and tea consumption, suggesting that these beverages may differentially influence brain connectivity patterns, potentially acting as a mediating factor for the risk of cognitive decline and AD.

Our findings regarding coffee consumption demonstrate that higher intake correlates positively with increased connectivity across multiple neural networks, including the Motor Execution Network (IC 11), Sensorimotor Network (IC 12), Fronto-Cingular Network (IC 14), and Prefrontal and ‘What’ Pathway Network (IC 21). These results align with previous studies suggesting the potential neuroprotective effects of coffee consumption [20,21,24].

The neuroprotective effects of coffee may be attributed to its rich polyphenol content, particularly chlorogenic acid. Chlorogenic acid, one of the most prevalent polyphenols in the diets of coffee drinkers, is found in both caffeinated and decaffeinated coffee, with amounts ranging from 70 to 350 mg per cup [51,52]. Its antioxidant properties contribute to reducing oxidative stress [53], which may act as a protective mechanism against neuronal injury, according to recent research [50]. Other beneficial compounds in coffee include caffeic acid, which is known for its anti-inflammatory and anti-carcinogenic properties [54], and trigonelline, the second most abundant alkaloid, which may offer neuroprotection and potential benefits in managing neurodegenerative conditions (like AD) [55].

Interestingly, our study revealed that the APOE genotype and family history of AD play a crucial role in modulating coffee’s effect on the Memory Consolidation Network. One study suggested that in the earliest stages of AD, functional connectivity between the medial temporal lobe and the anterior temporal system decreases before any disruption occurs in its connectivity with the posterior medial system [56]. For APOE4 carriers and those with a family history of AD, increased coffee consumption was associated with less Memory Consolidation Network connectivity. Conversely, APOE4 non-carriers and those without a family history of AD showed more connectivity in this network for higher coffee intake.

It is worth noting that previous studies have shown inconsistent results concerning coffee consumption and dementia risk [19,20,21,57,58]. A potential explanation for these discrepancies could be the lack of differentiation between coffee types in some studies [19]. Our study specifically focused on filtered coffee, which has also been associated with a decreased dementia risk in another recently published study [19].

With respect to tea consumption, our results reveal intriguing differences between tea types. Green tea consumption was associated with increased connectivity in the Extrastriate Visual Network (IC 4) and Primary Visual Network (IC 8). Conversely, the higher consumption of standard tea (typically black tea in the UK) was linked to decreased connectivity across several networks, including the Memory Consolidation Network (IC 7), Motor Execution Network (IC 11), Fronto-Cingular Network (IC 14), and Prefrontal and ‘What’ Pathway Network (IC 21). Interestingly, the APOE4 genotype emerged as an important mediating factor in these associations. Specifically, APOE4-positive participants showed significantly reduced neural network connectivity in the Sensorimotor Network (IC 12) with increased standard tea consumption.

These findings contribute to the ongoing debate about tea’s neuroprotective effects. While some studies support the neural protective effects of green tea/standard tea [59,60,61], others suggest no association between tea consumption and dementia risk [19]. Our results highlight the importance of distinguishing between tea types in such analyses.

The differential effects observed between green tea and standard tea may be attributed to their distinct processing methods and resulting bioactive compounds. Green tea, prepared by dehydrating tea leaves without oxidation, retains high concentrations of monomeric polyphenols from the catechins group, such as epigallocatechin-3-gallate (EGCG). Green tea contains several bioactive compounds that may contribute to its potential neuroprotective effects: catechins, which possess antioxidative and anti-inflammatory properties, may help to reduce AD risk [62]; theanine may reduce psychosocial stress [63] and influences glutamate levels in the brain [64]; and thirdly, Pyrroloquinoline Quinone (PQQ) shows potential in reducing neurodegeneration caused by oxidative stress [65]. In contrast, black tea undergoes fermentation, leading to the oxidation of these compounds [66].

The negative effects associated with standard (i.e., black) tea consumption in our study align with some previous research. A study by Dr. Sun and colleagues [25] suggested that excessive tea consumption may increase the risk of AD, correlating with decreased gray matter and the right hippocampus volume. However, it is important to note that their study had some methodological uncertainties regarding tea consumption variables. The authors indicated that tea consumption was measured based on the UKB Data-field ID: 100309, but this ID is not a valid indicator of tea consumption [25].

A Japanese study by Noguchi-Shinohara et al. [67] found that the consumption of green tea was associated with a significantly lower risk of cognitive decline, whereas no such association was found for black tea. Similarly, a US-based prospective study found no effects of black tea consumption on cognitive decline among older men (aged 65 +) [68]. The mechanisms and causes of the potential negative effects of black tea/UK standard tea are not fully understood and need more investigation; however, possible factors could include the oxidation of polyphenols during fermentation, differences in the bioavailability of compounds, or interactions with other dietary components typically consumed with black tea in the UK (e.g., milk and sugar).

Our study had several limitations. First, the self-reported nature of dietary data from the UK Biobank introduces the potential for recall bias and inaccuracies in quantifying consumption. While we adjusted for several confounding variables, such as age, sex, and socioeconomic status, unmeasured factors—such as personality traits, overall diet quality, and specific beverage preparation methods—may still have influenced the observed associations. For instance, adding milk or sugar to black tea could alter the bioavailability of bioactive compounds. It is also important to highlight that the study was conducted by associations using LMMs and that causal inference cannot be inferred. While studies in rodent models provide mechanistic insights into the effects of coffee and tea compounds on cognitive function [69], their findings must be interpreted cautiously when extrapolated to humans due to differences in metabolism and administration methods [70,71].

Second, while this study focused on broad APOE-based groupings (e.g., APOE4-positive vs. APOE4-negative individuals), there was no sufficient sample size to examine specific genotypes such as homozygous APOE4 (i.e., E4/E4) or variants related to APOE2 [72]. Moreover, it was not feasible to conduct subgroup genotype analyses and elevate Type I errors. Future research with larger sample sizes may be able to stratify by genotype and analyze nutrition data with brain and cognitive outcomes.

Third, the generalization of the findings is largely limited to people of European ancestry. The predominantly White UK Biobank cohort (94.84% of participants) limits the applicability of these results to other ethnic and cultural groups, especially given the variation in tea and coffee consumption habits, genetic predispositions, and dietary patterns across populations. In a related fashion, the UK Biobank does not distinguish between subtypes of tea or coffee or ways of preparing it. Nonetheless, dietary practices common in the UK, such as standard black tea consumption with milk and sugar, may differ significantly from those in other regions. Future studies should aim to include more ethnically and culturally diverse cohorts to explore population-specific differences in the relationships between beverage consumption, genetic predispositions, and neural connectivity.

## 5. Conclusions

This study provides novel insights into the differential effects of coffee and tea consumption on neural network connectivity, considering the potential mediating effects related to genetic and familial risk factors for AD. Our findings suggest that coffee intake generally correlates with increased neural network connectivity, while standard tea consumption is associated with reductions in the connectivity of neural networks. Importantly, these relationships are modulated by APOE4 status and a family history of AD. The contrasting effects observed between green and black tea highlight the need for more nuanced and detailed research in this area. Lastly, these results underscore the complex interplay between dietary habits, genetic factors, and brain function, emphasizing the importance of personalized approaches in understanding and potentially mitigating the risk of cognitive decline.

## Figures and Tables

**Table 1:** Demographic characteristics of participants.

Characteristic		
Baseline age, mean (SD), y	55.07 (7.48)	Range: 40–70
Body mass index (BMI), mean (SD), kg/m^2^	26.59 (4.17)	Range: 14.74–56.12
Women, %	52.54	
APOE ε4 status, %	27.68	
Family history of AD, %	24.26	
Smoking status, %		
Never	60.74	
Previous	32.89	
Current	6.37	
Alcohol status, %		
Never	2.45	
Previous	1.95	
Current	95.60	
Filtered coffee, mean (SD), cups/250 mL	1.53 (0.87)	Range: 0–6
Standard tea, mean (SD), cups/250 mL	3.1 (1.47)	Range: 0–6
Green tea, mean (SD), cups/250 mL	1.41 (1.01)	Range: 0–6

AD = Alzheimer’s disease; APOE = apolipoprotein E. All measures were obtained at baseline with the exception of tea and coffee intake (the average over five visits).

**Table 2:** Estimates of main effects for coffee and standard tea and green tea.

Neural Network	Filtered Coffee			Standard Tea			Green Tea		
	Beta	SE	*p* Value	Beta	SE	*p* Value	Beta	SE	*p* Value
IC 4	**0.0657**	**0.0168**	**0.0001**	−0.0109	0.0068	0.1122	**0.0512**	**0.0248**	**0.0396**
IC 7	0.0095	0.0129	0.4626	**−0.0105**	**0.0049**	**0.0329**	−0.0016	0.0170	0.9245
IC 8	**0.0773**	**0.0265**	**0.0036**	−0.0058	0.0104	0.5736	**0.0782**	**0.0377**	**0.0385**
IC 11	**0.0240**	**0.0121**	**0.0471**	**−0.0119**	**0.0047**	**0.0111**	0.0098	0.0161	0.5438
IC 12	**0.0431**	**0.0131**	**0.0011**	−0.0081	0.0052	0.1195	0.0090	0.0189	0.6341
IC 14	**0.0243**	**0.0084**	**0.0037**	**−0.0070**	**0.0033**	**0.0343**	−0.0045	0.0115	0.6979
IC 21	**0.0230**	**0.0109**	**0.0340**	**−0.0097**	**0.0043**	**0.0227**	−0.0082	0.0149	0.5814

Bold text denotes *p* < 0.05.

**Table 3:** Estimates for coffee and standard tea interactions by risk factors.

Neural Network	Filtered Coffee				Standard Tea			
	APOE4-Negative		APOE4-Positive		APOE4-Negative		APOE4-Positive	
	Beta	*p*-Value	Beta	*p*-Value	Beta	*p*-Value	Beta	*p*-Value
IC4	0.0704	0.0000	0.0667	0.0001	−0.0150	0.0009	−0.0030	0.6662
IC7	**0.0302**	**0.0002**	**−0.0313**	**0.0099**	−0.0126	0.0001	−0.0046	0.3483
IC8	0.0876	0.0000	0.0973	0.0001	−0.0073	0.2794	−0.0040	0.6964
IC11	0.0210	0.0077	0.0512	0.0000	−0.0102	0.0011	−0.0219	0.0000
IC12	0.0348	0.0000	0.0787	0.0000	**−0.0034**	**0.3188**	**−0.0261**	**0.0000**
IC14	0.0313	0.0000	0.0211	0.0154	−0.0109	0.0000	0.0018	0.6042
IC21	0.0322	0.0000	0.0127	0.2278	−0.0064	0.0205	−0.0195	0.0000

Bold text denotes *p* < 0.05.

## Data Availability

The data in this study are owned by the UK Biobank (www.ukbiobank.ac.uk (accessed on 11 August 2024)), a data repository that can be accessed by applying through the UK Biobank Access Management System (www.ukbiobank.ac.uk/register-apply (accessed on 11 August 2024)). Due to the legal agreement, as researchers, we do not have permission to share the data, and we are not entitled to republish or otherwise make available any UK Biobank data at the individual participant level. All analyses and intellectual content separate from UK Biobank are available on a request basis.

## Supplementary Materials

The following supporting information can be downloaded at https://www.mdpi.com/article/10.3390/nu16244303/s1. Table S1: Interpretation of independent components that constitute neural networks in UK Biobank.
