# Supplementary material for: Associations of Coffee and Tea Consumption on Neural Network Connectivity: Unveiling the Role of Genetic Factors in Alzheimer’s Disease Risk
Source: Nutrients. 2024 Dec 13;16(24):4303. doi: 10.3390/nu16244303 (PMC11677865; doi:10.3390/nu16244303)
Supplement: Supplementary file 1 [file nutrients-16-04303-s001.zip › nutrients-3333547-supplementary.pdf]

Supplementary Table S1. Interpretation of Independent Components that Constitute Neural Networks in UK Biobank

Independent Component	Neural Network Description
IC 4	Extrastriate Visual Network
IC 7	Memory Consolidation Network
IC 8	Primary Visual Network
IC 11	Motor Execution Network
IC 12	Sensorimotor Network
IC 14	Fronto-Cingular Network
IC 21	Prefrontal and 'What' Pathway Network

IC4 was composed of extrastriate regions with some impingement on primary visual areas, suggesting a network focused on multi-modal visual processing.

IC7 was a memory consolidation network composed of activation in precuneus, posterior cingulate, and lateral parietal cortices that streamed down to medial temporal lobe including parahippocampal gyrus and entorhinal cortex, with little activity in hippocampus proper.

IC8 showed activation exclusively in posteromedial occipital gyrus, suggesting primary visual processing in V1 rather than extrastriate areas seen in IC4.

IC11 was a motor execution network comprised almost exclusively of pre-central gyrus activity, thalamus, and both anterior and posterior medial cerebellum.

IC12 was a sensorimotor network that almost exclusively included pre- and post-central gyri.

IC14 was a fronto-cingular network composed of all segments of cingulate gyrus except for the rostrum, as well as medial frontal gyrus.

IC21 was a complex neural network to interpret, with activation hot-spots predominantly in bilateral inferior frontal gyrus, as well as left superior mediofrontal and orbitofrontal gyri, precuneus, and left medial temporal cortex. This network may involve top-down cognitive processing of object stimuli in the 'what' pathway.

Supplementary R Codes

```
library(dplyr)

#main effects test for tea_coffee data

library(lmerTest)

df<-read.csv(file = 'C:\\****\\tea_coffee.csv')

# Fit the model

df$APOE_all <- as.factor(df$APOE_all)

df$family_history_AD <- as.factor(df$family_history_AD)

df$Sex <- as.factor(df$Sex)

df$AlcoholY08 <- as.factor(df$AlcoholY08)

df$SmokingY08 <- as.factor(df$SmokingY08)

df$Assessment_0 <- as.factor(df$Assessment_0)

results <- data.frame()

# List of variables to test

test_vars <- c("FilterCoffee_mean", "StandTea_mean", "GreenTea_mean")


dependent_vars <- c("rsfMRI_Component4", "rsfMRI_Component7","rsfMRI_Component8",
"rsfMRI_Component11", "rsfMRI_Component12", "rsfMRI_Component14",
"rsfMRI_Component21")


# Double loop through each dependent and test variable and fit the model

for (dep_var in dependent_vars) {

  for (test_var in test_vars) {


    # Construct the formula

    formula <- as.formula(paste(dep_var, "~ APOE_all + family_history_AD + Age_Baseline +
Sex +

                              BMI_Y08andY12andY14_mean + AlcoholY08 + SmokingY08 + Townsend
+", test_var, "+

                              (1 | Assessment_0)"))
```

````
    # Fit the model
    model <- lmer(formula, data = df, REML=FALSE)

   # Get the summary
    model_summary <- summary(model)

    # Extract the results for the test variable
    estimate <- model_summary$coefficients[test_var, "Estimate"]
    std_error <- model_summary$coefficients[test_var, "Std. Error"]
    p_value <- model_summary$coefficients[test_var, "Pr(>|t|)"]

    # Add the results to the data frame
    results <- rbind(results, data.frame(Dependent_Variable = dep_var,
                                         Test_Variable = test_var,
                                         Estimate = estimate,
                                         Std_Error = std_error,
                                         p_value = p_value))
  }
}
write.csv(results, "C:\\***\\maineffect.csv",na='',row.names=FALSE)
rm(list=ls())
```
```{r}
#interaction test for tea coffee data
library(lmerTest)
df<-read.csv(file = 'C:\\***\\tea_coffee.csv')
````

```
# Fit the model -- factor all factor variables

factor_vars <- c("AlcoholY08", "SmokingY08", "Assessment_0", "EID")

for (var in factor_vars) {

  df[[var]] <- as.factor(df[[var]])

}

rm(factor_vars,var)


# Scale all continuous variables

scale_vars <- c("GreenTea_mean", "FilterCoffee_mean",
"StandTea_mean","rsfMRI_Component4","rsfMRI_Component7",

                "rsfMRI_Component8", "rsfMRI_Component11", "rsfMRI_Component12",
"rsfMRI_Component14", "rsfMRI_Component21")


for (var in scale_vars) {

  df[[var]] <- as.numeric(df[[var]])

}

rm(scale_vars, var)


# Define the dependent variables

dependent_vars <- c("rsfMRI_Component4","rsfMRI_Component7","rsfMRI_Component8",
"rsfMRI_Component11", "rsfMRI_Component12", "rsfMRI_Component14",
"rsfMRI_Component21")


# List of variables to test

test_vars <- c("FilterCoffee_mean", "StandTea_mean", "GreenTea_mean")


# List of factors

factor_vars <- c("APOE_all", "family_history_AD")


# Initialize a results data frame
```

```
results <- data.frame()

# Loop over each dependent variable
for(factor_var in factor_vars) {
 for (dep_var in dependent_vars) {
  for (test_var in test_vars) {

   # Construct the formula
   formula <- as.formula(paste(dep_var, "~ APOE_all +  family_history_AD + Age_Baseline +
Sex +
                              BMI_Y08andY12andY14_mean + AlcoholY08 + SmokingY08 + Townsend
+",test_var,"+", factor_var, ":", test_var, "+
                              (1 | Assessment_0)"))

   # Fit the model
   model <- lmer(formula, data = df, REML=FALSE)

 # Get the summary
   model_summary <- summary(model)

   # Extract the results for the interaction term
   interaction_term <- paste(factor_var, test_var, sep=":")
   estimate <- model_summary$coefficients[interaction_term, "Estimate"]
   std_error <- model_summary$coefficients[interaction_term, "Std. Error"]
   p_value <- model_summary$coefficients[interaction_term, "Pr(>|t|)"]

   # Add the results to the data frame
   results <- rbind(results, data.frame(Dependent_Variable = dep_var,
                                          Factors = factor_var,
```

```
                                Test_Variable = test_var,
                                Estimate = estimate,
                                Std_Error = std_error,
                                P_Value = p_value))
    }
  }
}
write.csv(results, "C:\\***\\interactions.csv",na='',row.names=FALSE)
rm(list=ls())
```

```{r}
#step 2 for interaction coffee
# Load libraries
library(lme4)
library(lmerTest)  # Provides p-values for fixed effects
library(dplyr)

# Load the dataset (replace 'your_dataset.csv' with your actual dataset)
df <- read.csv(file = 'C:\\***\\tea_coffee.csv')

# List of required columns for the analysis
required_columns_coffee <- c("rsfMRI_Component7", "rsfMRI_Component11",
"rsfMRI_Component14",
                        "rsfMRI_Component21", "rsfMRI_Component12", "Assessment_0",
"APOE_all",
                         "family_history_AD", "Age_Baseline", "Sex", "SmokingY08",
                        "AlcoholY08", "BMI_Y08andY12andY14_mean", "Townsend",
"FilterCoffee_mean")
```

```
# Ensure that the required columns exist in the dataset
missing_columns <- setdiff(required_columns_coffee, colnames(df))
if (length(missing_columns) > 0) {
  stop(paste("The following required columns are missing from the dataset:",
paste(missing_columns, collapse = ", ")))
}

# Create a complete dataset by removing rows with NA in the required columns
df <- df[complete.cases(df[, required_columns_coffee]), ]

# Ensure that factors are treated correctly
df$APOE_all <- as.factor(df$APOE_all)
df$family_history_AD <- as.factor(df$family_history_AD)
df$Assessment_0 <- as.factor(df$Assessment_0)  # For random effects

# List of rsfMRI components to loop through
rsfMRI_components <- c("rsfMRI_Component7", "rsfMRI_Component11",
"rsfMRI_Component14",
                       "rsfMRI_Component21", "rsfMRI_Component12")

# Initialize an empty data frame to store results
all_results_df <- data.frame()

### Loop 1: Testing by family_history_AD ###
for (component in rsfMRI_components) {
  # Check if the component exists in the dataset
  if (!component %in% colnames(df)) {
```

```
      warning(paste("Component", component, "is missing in the dataset. Skipping this
component."))
      next
    }

    # Dynamically update the formula for each component
    formula <- as.formula(paste(component, "~ FilterCoffee_mean + (1 | Assessment_0)"))

    # Loop over family_history_AD
    for (family_history in unique(df$family_history_AD)) {
      # Subset the data for the current family_history_AD
      df_subset <- subset(df, family_history_AD == family_history)

      if (nrow(df_subset) > 0) {
        # Fit the mixed model
        model <- lmer(formula, data = df_subset, REML = FALSE)  # Use ML method
        summary_model <- summary(model)$coefficients
      } else {
        summary_model <- data.frame(Estimate = NA, `Std. Error` = NA, `Pr(>|t|)` = NA)
      }

      # Filter for "FilterCoffee_mean" term only (exclude intercept)
      summary_model <- summary_model[rownames(summary_model) == "FilterCoffee_mean",
, drop = FALSE]

      # Create a data frame to store results for the current family_history_AD
      results_df <- data.frame(
        rsfMRI_Component = rep(component, nrow(summary_model)),
        Variable = "family_history_AD",
```

```
      Group = rep(family_history, nrow(summary_model)),
      Term = rownames(summary_model),
      Estimate = summary_model[, "Estimate"],
      Std_Error = summary_model[, "Std. Error"],
      p_value = summary_model[, "Pr(>|t|)"]
    )

    # Combine the current results with the overall results
    all_results_df <- rbind(all_results_df, results_df)
  }
}

### Loop 2: Testing by APOE_all ###
for (component in rsfMRI_components) {
  # Check if the component exists in the dataset
  if (!component %in% colnames(df)) {
    warning(paste("Component", component, "is missing in the dataset. Skipping this
component."))
    next
  }

  # Dynamically update the formula for each component
  formula <- as.formula(paste(component, "~ FilterCoffee_mean + (1 | Assessment_0)"))

  # Loop over APOE_all
  for (APOE in unique(df$APOE_all)) {
    # Subset the data for the current APOE_all
    df_subset <- subset(df, APOE_all == APOE)
```

```
    if (nrow(df_subset) > 0) {
      # Fit the mixed model
      model <- lmer(formula, data = df_subset, REML = FALSE)  # Use ML method
      summary_model <- summary(model)$coefficients
    } else {
      summary_model <- data.frame(Estimate = NA, `Std. Error` = NA, `Pr(>|t|)` = NA)
    }

    # Filter for "FilterCoffee_mean" term only (exclude intercept)
    summary_model <- summary_model[rownames(summary_model) == "FilterCoffee_mean",
, drop = FALSE]

    # Create a data frame to store results for the current APOE_all
    results_df <- data.frame(
      rsfMRI_Component = rep(component, nrow(summary_model)),
      Variable = "APOE_all",
      Group = rep(APOE, nrow(summary_model)),
      Term = rownames(summary_model),
      Estimate = summary_model[, "Estimate"],
      Std_Error = summary_model[, "Std. Error"],
      p_value = summary_model[, "Pr(>|t|)"]
    )

    # Combine the current results with the overall results
    all_results_df <- rbind(all_results_df, results_df)
  }
}

# Write the combined results to a CSV file
```

```
write.csv(all_results_df, "C:\\***\\interactions_Coffee_all_components.csv",
          na = '', row.names = FALSE)
rm(list = ls())
```

```{r}
#step 2 for interaction tea
# Load libraries
library(lme4)
library(lmerTest)  # Provides p-values for fixed effects
library(dplyr)

# Load the dataset (replace 'your_dataset.csv' with your actual dataset)
df <- read.csv(file = 'C:\\***\\tea_coffee.csv')

# List of required columns for the analysis
required_columns_tea <- c("rsfMRI_Component7", "rsfMRI_Component11",
"rsfMRI_Component14",
                          "rsfMRI_Component21", "rsfMRI_Component12", "Assessment_0",
"APOE_all",
                          "family_history_AD", "Age_Baseline", "Sex", "SmokingY08",
                          "AlcoholY08", "BMI_Y08andY12andY14_mean", "Townsend",
"StandTea_mean")

# Ensure that the required columns exist in the dataset
missing_columns <- setdiff(required_columns_tea, colnames(df))
if (length(missing_columns) > 0) {
  stop(paste("The following required columns are missing from the dataset:",
paste(missing_columns, collapse = ", ")))
}
```

```
# Create a complete dataset by removing rows with NA in the required columns
df <- df[complete.cases(df[, required_columns_tea]), ]

# Ensure that factors are treated correctly
df$APOE_all <- as.factor(df$APOE_all)
df$family_history_AD <- as.factor(df$family_history_AD)
df$Assessment_0 <- as.factor(df$Assessment_0)  # For random effects

# List of rsfMRI components to loop through
rsfMRI_components <- c("rsfMRI_Component7", "rsfMRI_Component11",
"rsfMRI_Component14",
                       "rsfMRI_Component21", "rsfMRI_Component12")

# Initialize an empty data frame to store results
all_results_df <- data.frame()

### Loop 1: Testing by family_history_AD ###
for (component in rsfMRI_components) {
  # Check if the component exists in the dataset
  if (!component %in% colnames(df)) {
    warning(paste("Component", component, "is missing in the dataset. Skipping this
component."))
    next
  }

  # Dynamically update the formula for each component
  formula <- as.formula(paste(component, "~ StandTea_mean + (1 | Assessment_0)"))
```

```
  # Loop over family_history_AD
  for (family_history in unique(df$family_history_AD)) {
    # Subset the data for the current family_history_AD
    df_subset <- subset(df, family_history_AD == family_history)

    if (nrow(df_subset) > 0) {
      # Fit the mixed model
      model <- lmer(formula, data = df_subset, REML = FALSE)  # Use ML method
      summary_model <- summary(model)$coefficients
    } else {
      summary_model <- data.frame(Estimate = NA, `Std. Error` = NA, `Pr(>|t|)` = NA)
    }

    # Filter for "StandTea_mean" term only (exclude intercept)
    summary_model <- summary_model[rownames(summary_model) == "StandTea_mean", ,
drop = FALSE]

    # Create a data frame to store results for the current family_history_AD
    results_df <- data.frame(
      rsfMRI_Component = rep(component, nrow(summary_model)),
      Variable = "family_history_AD",
      Group = rep(family_history, nrow(summary_model)),
      Term = rownames(summary_model),
      Estimate = summary_model[, "Estimate"],
      Std_Error = summary_model[, "Std. Error"],
      p_value = summary_model[, "Pr(>|t|)"]
    )

    # Combine the current results with the overall results
```

```
      all_results_df <- rbind(all_results_df, results_df)
   }
}

### Loop 2: Testing by APOE_all ###
for (component in rsfMRI_components) {
   # Check if the component exists in the dataset
   if (!component %in% colnames(df)) {
      warning(paste("Component", component, "is missing in the dataset. Skipping this component."))
      next
   }

   # Dynamically update the formula for each component
   formula <- as.formula(paste(component, "~ StandTea_mean + (1 | Assessment_0)"))

   # Loop over APOE_all
   for (APOE in unique(df$APOE_all)) {
      # Subset the data for the current APOE_all
      df_subset <- subset(df, APOE_all == APOE)

      if (nrow(df_subset) > 0) {
         # Fit the mixed model
         model <- lmer(formula, data = df_subset, REML = FALSE)  # Use ML method
         summary_model <- summary(model)$coefficients
      } else {
         summary_model <- data.frame(Estimate = NA, `Std. Error` = NA, `Pr(>|t|)` = NA)
      }
```

```
        # Filter for "StandTea_mean" term only (exclude intercept)
        summary_model <- summary_model[rownames(summary_model) == "StandTea_mean",
drop = FALSE]

        # Create a data frame to store results for the current APOE_all
        results_df <- data.frame(
          rsfMRI_Component = rep(component, nrow(summary_model)),
          Variable = "APOE_all",
          Group = rep(APOE, nrow(summary_model)),
          Term = rownames(summary_model),
          Estimate = summary_model[, "Estimate"],
          Std_Error = summary_model[, "Std. Error"],
          p_value = summary_model[, "Pr(>|t|)"]
        )

        # Combine the current results with the overall results
        all_results_df <- rbind(all_results_df, results_df)
    }
}

# Write the combined results to a CSV file
write.csv(all_results_df, "C:\\***\\interactions_Tea_all_components.csv",
          na = '', row.names = FALSE)
rm(list = ls())
```
